# ASSOCIATION BETWEEN NEIGHBORHOOD DEPRIVATION AND PLACE OF DEATH: A POPULATION STUDY

**DOI:** 10.1093/geroni/igad104.2037

**Published:** 2023-12-21

**Authors:** Harriet Mather

**Affiliations:** Icahn School of Medicine at Mount Sinai, New York City, New York, United States

## Abstract

While recent decades have witnessed an overall trend away from deaths in hospitals and facilities and towards deaths at home, socioeconomic disparities persist. Minoritized individuals while less likely to die in a nursing facility are more likely to die in hospital and (except for dementia) less likely to die at home; more educated individuals are also less likely to die in a nursing facility but conversely less likely to die in hospital and more likely to die at home. Despite growing interest in the role of neighborhood-level factors in health disparities, no studies have examined the impact of neighborhood deprivation on place of death. Using a representative sample of Medicare beneficiaries (n = 3043) drawn from the National Health and Aging Trends Study, we examined the association between census-tract level social deprivation index (quartiles) and place of death (home vs all else, hospital vs all else, nursing facility vs all else, hospice residence vs all else) in a series of multivariable regression models, adjusting for relevant sociodemographic (age, sex, race, education, income) and clinical covariates (dementia, heart disease, lung disease, cancer). Compared with residence in the least deprived quartile, residence in a neighborhood in the most deprived quartile was associated with reduced odds of home death (aOR: 0.66 (0.45, 0.99)) and increased odds of death in a nursing facility (aOR: 1.68 (1.00, 2.80)). Future studies should confirm these findings and examine mechanisms through which neighborhood deprivation impacts place of death (including availability and quality of home-based clinical care.

